# Magnetic Sphincter Augmentation for Gastroesophageal Reflux After Sleeve Gastrectomy: A Systematic Review

**DOI:** 10.1007/s11695-024-07523-8

**Published:** 2024-10-15

**Authors:** Francesco Cammarata, Martina Novia, Alberto Aiolfi, Riccardo Damiani, Michele Manara, Alessandro Giovanelli, Rossana Daniela Berta, Marco Anselmino, Cristina Ogliari, Davide Bona, Luigi Bonavina

**Affiliations:** 1 IRCCS Ospedale Galeazzi–Sant’Ambrogio, Milan, Italy; 2 University of Milan, Milan, Italy; 3 IRCCS Policlinico San Donato, San Donato Milanese, Italy

**Keywords:** Gastroesophageal reflux, Refractory GERD, Bariatric surgery, Sleeve gastrectomy, Magnetic sphincter augmentation, LINX procedure, Roux-en-Y gastric bypass

## Abstract

This systematic review aims to evaluate the current evidence regarding safety and efficacy of magnetic sphincter augmentation (MSA) for the treatment of gastroesophageal reflux disease (GERD) after laparoscopic sleeve gastrectomy (LSG). Conversion to Roux-en-Y gastric bypass (RYGB) carries the risk of surgical and metabolic complications and may be contraindicated in patients with normalized or near-normalized body mass index. The LINX™ procedure aims to restore LES competency and to repair the crura. We included 109 patients (14 studies) undergoing LINX™ implant after LSG. Median follow-up was 18.9 months (range 0.3–63). Both the GERD-HRQL (38 ± 13 vs. 10 ± 11; *p* = 0.0078) and daily PPI use (97.4% vs. 25.3%; *p* < 0.0001) were significantly improved. Overall, 31.8% of the patients experienced device-related adverse events, mainly self-limiting. LINX™ explant for esophageal erosion occurred in 0.9% of the patients.

**Supplementary Information:**

The online version contains supplementary material available at 10.1007/s11695-024-07523-8.

## Introduction

Gastroesophageal reflux disease (GERD) poses a significant clinical challenge after laparoscopic sleeve gastrectomy (LSG), highlighting the need for effective and standardized treatment options. A meta-analysis by Yeung et al. indicated that de novo GERD, esophagitis, and Barrett’s esophagus may develop in up to 23%, 30%, and 6% of the patients after LSG, respectively [1]. Furthermore, both pre-existing and de novo GERD have been associated with HH and intrathoracic sleeve migration in up to 30% of the cases [2–4]. Anatomical and physiological changes associated with LSG may result in a regurgitation-dominant GERD phenotype unresponsive to conventional medical treatment [5]. Conversion to laparoscopic Roux-en-Y gastric bypass (RYGB) is generally considered for patients with inadequate excess weight loss or weight regain, but a high proportion of patients refuse this operation due to the major anatomical changes involved and the perceived side-effects [6, 7]. In addition, it is still controversial whether class 1 obesity (BMI > 30 < 35) patients may still benefit from conventional antireflux surgery rather than undergoing upfront RYGB.

The magnetic sphincter augmentation (MSA) device has become a promising alternative to traditional fundoplication for the treatment of primary GERD, but it is not approved yet for routine use as prophylactic or therapeutic intervention in the bariatric population [8–12]. The purpose of this review was to analyze the current literature and assess safety, feasibility, and efficacy of the MSA (LINX™ procedure) for the treatment of post-LSG GERD.

## Materials and Methods

### Study Design and Review Process

A systematic review was reported following the Preferred Reporting Items for Systematic Reviews and Meta-Analyses checklist guideline (PRISMA 2020) [13]. Since the study included a review of published articles and study-level results, institutional review board approval or exemption was not required. Study was registered with PROSPERO (CRD42024572025).

### Data Sources and Search Strategy

We conducted a literature search on the use of MSA in GERD patients after LSG across all published studies. The last search date was May 1, 2024. The databases consulted included Embase, Web of Science, Scopus, PubMed, Cochrane Library, and Google Scholar [14]. The comprehensive search strategy included a combination of the following keywords, synonyms, and Medical Subject Headings (MeSH) terms: (“LINX” OR “MSA” OR (“Magnetic” AND “Sphincter”) OR (“Magnetic” AND “Sphincter” AND “Augmentation”)) AND (“Sleeve” OR “Sleeve gastrectomy” OR “Gastroplasty” OR “Magenstrasse”). The search strategy is depicted in Appendix 1. After screening all the articles from the database searches, we reviewed the reference lists of the articles to identify any additional potential reference that may have been missed.

### Selection Criteria

Inclusion criteria: (a) clinical studies reporting the use of LINX™ procedure in adult patients (> 18 years) who previously underwent LSG; (b) when two or more articles were published by the same institution, study group, or used the same dataset, the articles with the longest follow-up or the largest sample size were included in the review; (c) in cases of duplicate studies with overlapping patient data, only the most comprehensive reports were included for analysis. Exclusion criteria: (a) not English-written articles; (b) studies reporting mixed data that included other bariatric surgical procedures, where data specific to LSG patients could not be accurately discerned; (c) abstracts, conference papers, and review articles were not considered for analysis.

### Screening, Selection, and Data Extraction

All titles and abstract retrieved from databases were initially screened independently for eligibility by two authors (MN and FC), based on the inclusion and exclusion criteria. After duplicates and ineligible articles’ removal, a second round of screening using the same criteria was conducted via full-text review of the remaining articles. Data extracted in a datasheet included study characteristics (first author name, year, and country), number of patients, time frame, clinical and demographic characteristics of the patient population, operative data, and postoperative clinical outcomes. All data were computed independently by two investigators (MN and FC) and compared at the end of the review process. A third author (AA) reviewed the database and determined discrepancies.

### Quality Assessment

Three authors (MN, FC, and MM) independently assessed the methodologic quality of the selected studies using both the ROBINS-I tool for observational studies and the Murad et al. tool for case reports and case series [15, 16]. All selected studies were then graded as having low, moderate, high, or unclear risk of bias.

### Outcomes of Interest and Data Analysis

The primary study aim was to evaluate the effectiveness and safety of MSA placement in the treatment of GERD in post-LSG patients. Objective outcomes included the reduction in GERD symptoms, reduction in the use of PPIs, reduction of esophageal acid exposure, and occurrence and type of adverse events. Whenever possible, data were combined and presented with a weighted mean and pooled standard deviation, as well as weighted average percentages. This approach allowed for a comprehensive and representative assessment of the data collected across multiple studies. Differences between pre- and posttreatment measures were evaluated using Student’s *t* test for continuous variables, the chi-square test for categorical variables, and McNemar’s test for paired nominal data when appropriate. A *p* value of less than 0.05 was considered statistically significant. The statistical analysis was performed using IBM SPSS Statistics 25.0.

## Results

### Literature Search and Quality Assessment

**Fig. 1:**
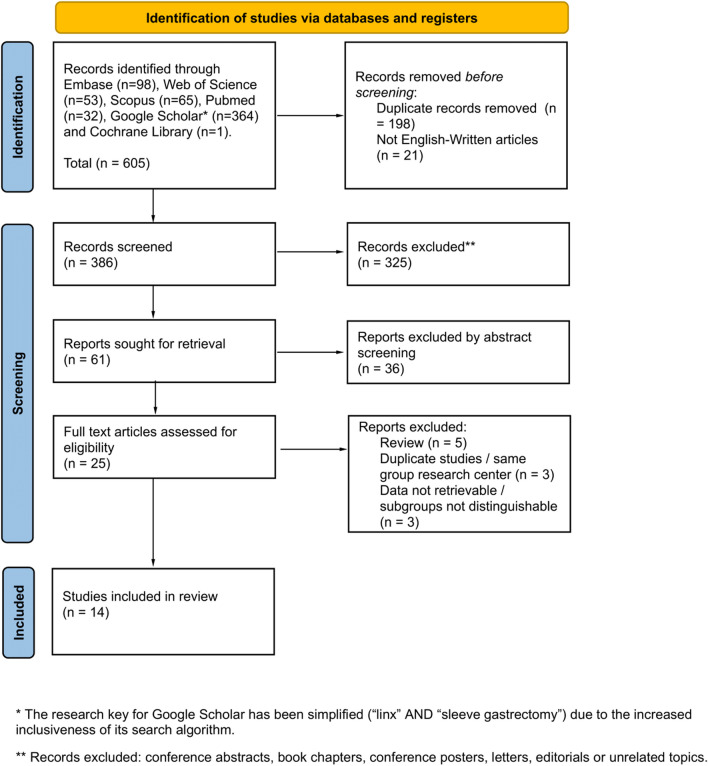
PRISMA 2020 flow diagram for new systematic reviews which included searches of databases and registers only

The literature search process is summarized in the PRISMA flowchart shown in Fig. 1. A total of 605 records were identified. After removing 198 duplicate records and 21 records due to language issues, 386 records remained for screening. During the screening phase, 325 records were excluded based on the criteria outlined in the study protocol. Out of the screened records, 61 reports were sought for retrieval. Further screening of the abstracts led to the exclusion of 36 reports. The remaining 25 full-text articles were assessed for eligibility. Finally, 14 studies (109 patients) met the inclusion criteria and were considered in the final review [8–11, 17–26]. These studies span from 2015 to 2024 and were conducted in various countries, with 96.3% of the patients treated in the USA. There were 7 case reports, 5 retrospective observational studies, and 2 prospective observational studies.

The results of all studies, including patients’ demographics, clinical characteristics, operative data, and outcomes are summarized in Table 1. The risk of bias of 3 out of the 14 studies was graded as high, while other studies had a moderate risk (Supplementary Tables 1-2).

**Table 1:** Clinical data and outcomes of patients undergoing minimally invasive MSA implant after sleeve gastrectomy

Author, year, country, study design	No., pts	Age, mean ± SD	Sex (M:F)	BMI, median (range)	PPI daily use	Months from SG to MSA, median (range)	Preop. GERD-HRQL score, mean ± SD	Endoscopic findings (HH/esophagitis)	HRM/AET
Peine et al., 2024, USA, Ret	5	55.6 ± 7.7	1:4	30 (26; 38)	5/5	36 (21; 53)	30.2 ± 9.7	HH 2.9 ± 0.9 cm/esophagitis nr	Normal/3 acidic, 2 nr
Soler-Silva et al., 2023, Spain, CR	1	38	1:0	nr	1/1	nr	nr	HH nr/EGJ type 2, LA grade C	nr/nr
Khaitan et al., 2023, USA, PrO	30	47.1 ± 12.1	3:27	30.5 (23.1; 34.9)	30/30	38.4	35.6 ± 9.7	HH 2.2 cm/esophagitis 63% (7 gr. A, 12 gr. B)	Normal/15.3
Hawasli et al., 2023, USA, Ret	17	54 ± 12	3:14	31 (13; 49)	nr	41 (3; 79)	46 ± 19	HH 82%/esophagitis 35%	nRr/nr
Patel et al., 2022, USA, Ret	22	46 ± 10.9	2:20	32.3 (25.1; 51.9)	21/22	43.6 (4.2; 124.4)	43.8 ± 11.3	HH 100%/esophagitis nr	nRn/49.8
Bellorin et al., 2021, USA, PrO	13	38 ± 8	3:10	26.6 (15.2; 38)	nr	12 (4; 36)	nr	HH nr/esophagitis 77% (4 gr. A, 2 gr. B, 4 gr. C)	nRr/nr
Asti et al., 2021, Italy, CR	1	57	0:1	24.8	1/1	48	31	HH 3.1 cm/esophagitis 0%	Ineffective esophageal dysmotility/21.8
Broderick et al., 2020, USA and Argentina, Ret	8	43.1 ± 8.9	1:7	28.05 (21.1; 33.6)	7/8	nr	25 (7 nr)	HH nr/esophagitis nr	nRr/nr
Ndubizu et al., 2020, USA, CR	1	37	0:1	30.6	1/1	12	45	HH yes, small/esophagitis nr	Transient LES relaxation/nr
Ajabshir et al., 2019, USA, CR	1	32	0:1	nr	nr	nr	nr	HH yes, small/mild esophagitis	nRr/nr
Hawasli et al., 2017, USA, CR	1	25	0:1	27.9	1/1	30	64	HH yes, small/esophagitis: yes	Normal/nr
Desart et al., 2015, USA, Ret	7	50.3	5:2	34.4	7/7	12 (7; 36)	17.1	HH no/esophagitis nr	Hypotensive LES/nr
Pixner et al., 2022, Germany, CR	1	nr	0:1	27	1/1	24	nr	HH yes, small/erosive gastritis	Normal/nr
Bona et al., 2022, Italy, CR	1	45	0:1	27.7	1/1	84	37	HH 2 cm/esophagitis LA gr. A	Hypotensive LES/nr

**Author, year, country, study design**	**DeMeester score before MSA, mean ± SD**	**Type of surgery**	**Surgery duration (min), mean ± SD**	**MSA size, median (range)**	**Crural repair**	**Length of stay, mean ± SD**	**Follow-up (months), mean ± SD**	**PPI off**	**Postop. GERD-HRQL score, mean ± SD**
Peine et al., 2024, USA, Ret	55.6 ± 28.57	VL	nr	nr	3/5	1	18.8 ± 15.3	3/5	9.6 ± 15.1
Soler-Silva et al., 2023, Spain, CR	nr	VL	nr	nr	nr	nr	12	nr	nr
Khaitan et al., 2023, USA, PrO	54.1 ± 21.6	VL	58.1 ± 23.3	15.5 (14; 17)	27/30	0.7 ± 0.6	12 (1 lost at FU)	22/26	8.1 ± 11.3
Hawasli et al., 2023, USA, Ret	48 ± 26	VL	83 ± 27	15 (13; 17)	17/17	1.2 ± 0.6	63 ± 31 (1 lost at FU)	3/14	12 months: 8 ± 7 5 years: 19 ± 14
Patel et al., 2022, USA, Ret	39.8 ± 20.8	VL	nr	16 (15; 17)	22/22	nr	9 ± 5.6	5/22	16.7 ± 12.8
Bellorin et al., 2021, USA, PrO	46 ± 18.1	R	79 ± 17.8	nr	13/13	1 (1; 3)	10 ± 5	12/13	nr
Asti et al., 2021, Italy, CR	55.7	VL	80	nr	1/1	nr	8	1/1	nr
Broderick et al., 2020, USA and Argentina, Ret	25.7 ± 9.5	VL	80	nr	8/8	1.38	20.75	7/8	8
Ndubizu et al., 2020, USA, CR	nr	VL	nr	14	1/1	nr	12	1/1	14
Ajabshir et al., 2019, USA, CR	nr	VL	nr	nr	nr	2	0.46	nr	nr
Hawasli et al., 2017, USA, CR	66.6	VL	47	14	1/1	1	0.33	nr	7
Desart et al., 2015, USA, Ret	56.6	VL	nr	nr	nr	nr	0.92	nr	5.1
Pixner et al., 2022, Germany, CR	15	VL	nr	nr	1/1	nr	9	nr	nr
Bona et al., 2022, Italy, CR	68.7	VL	45	15	1/1	nr	25	1/1	nr

*nr* not reported, *nRr* no results reported, *Ret* retrospective, *CR* case report, *PrO* prospective observational, *MSA* magnetic sphincter augmentation, *SG* sleeve gastrectomy, *SD* standard deviation, *M:F* male to female ratio, *BMI* body mass index, *PPI* proton-pump inhibitors, *GERD-HRQL* gastro-esophageal reflux disease health-related quality of life, *HH* hiatal hernia, *LA* Los Angeles classification, *HRM* high-resolution manometry

### Demographics and Clinical Characteristics

The age of patients ranged from 25 to 73 years and the majority were females (82.5%). The mean BMI of patients at presentation was 29.8 ± 4.8 and ranged from 21.1 to 51.9. Prior to MSA implant, the majority (97.4%) of patients were treated with PPIs. Eleven studies reported the time from LSG to MSA implant, which ranged from 7 to 124 months. The GERD-HRQL score was reported in 10 studies, and the baseline weighted mean score was 38.2 ± 13.6. Information regarding the presence of hiatal hernia was reported in 10 studies, but only a few provided data on the hernia size. Sleeve herniation through the hiatus was estimated to be present in 89.6% of the patients, with a mean size of 2.1 ± 0.7 cm. Esophagitis was reported in 10 studies and was present in 56.3% of the patients. The DeMeester score and the acid exposure time were reported in 11 and 3 studies, respectively. The DeMeester score values ranged from 20.4 to 96.7, and the weighted mean was 47.2 ± 22.3. The AET % time values ranged from 15.3 to 49.8 (weighted mean 29.7).

### Operative and Outcome Data

Thirteen studies reported a successful laparoscopic LINX™ procedure without conversions in all patients. A robotic approach was reported in one study (13 patients) [26]. In 7 studies, the mean duration of surgery was 70.3 ± 23.7 min. A crural repair was performed in 95% of the patients, but none of the studies specified the extent of mediastinal dissection and the length of intra-abdominal esophagus. The size of the MSA device was reported in 6 studies and ranged from 13 to 17 beads (median 15), but scant information was provided regarding the sizing technique and postoperative radiologic position. Overall postoperative 30-day morbidity and mortality were 21.97% and 0, respectively. The mean duration of hospitalization was 0.98 ± 0.5 days. The MSA-related adverse events and the reasons for device removal are reported in Table 2.

**Table 2:** Post-LSG LINX-related adverse events

	***N* = 91**
	29/91 (31.8%)
AEs, *n* (%) Dysphagia Pain Nausea Diarrhea Esophageal spasm Pneumothorax Stricture Reflux Broken device Erosion Others	10 (11%)^*^ 3 (3.3%) 2 (2.2%) 1 (1.1%) 1 (1.1%) 1 (1.1%) 2 (2.2%) 2 (2.2%) 4 (4.4%)^**^ 1 (1.1%)º 8 (8.8%)^¶^

The total number of AEs exceeds the number of patients experiencing them because each patient can have multiple AEs. However, it was not possible to identify which patients had multiple AEs.

^*^Three patients required device removal for dysphagia.

^**^Two of these patients with broken devices underwent device removal.

ºOne patient with dysphagia underwent endoscopic dilatation with subsequent erosion that required laparoscopic device removal.

One patient presented intrathoracic migration and underwent device removal.

### Follow-Up and Outcomes

The follow-up time was reported in all studies. However, only 7 studies had a median follow-up of at least 1 year (median 18.9 months, range 0.33–63). MSA-related adverse events were reported in 9 studies. Throughout the follow-up, 29 of 91 patients (31.8%) experienced one or more adverse events (Table 2). Seven patients (6.4%) underwent MSA removal due to dysphagia (*n *= 3), broken device without dislocation (*n* = 2), erosion (*n* = 1), or intrathoracic migration (*n* = 1). Two devices were removed within the first 30 days post implant, the others at a median of 5 months (range 4–56 months). Endoscopic pneumatic dilation for dysphagia was required in 3 patients (2.7%). Two of them were dilated at 3 weeks and 6 months, respectively, but timing of intervention was not reported for the last patient. Overall, endoscopic dilation was successful in 2 of the 3 patients. No multiple dilatations were reported. Nine studies reported complete cessation of PPI use in 60.4% of the patients, daily use in 25.3% of the patients, and intermittent use in 14.3% of the patients. There was a statistically significant reduction of daily PPI use compared to preoperative baseline (97.4% vs. 25.3%; *p* < 0.0001).

Both pre- and postoperative GERD-HRQL scores were reported in 8 studies (82 patients). Compared to baseline preoperative values, the weighted average postoperative GERD-HRQL score was significantly lower (38.2 ± 13.6 vs. 10.2 ± 11.1; *p* = 0.0078). For patients with a BMI < 35 (*n* = 26), the mean pre-MSA GERD-HRQL score was 37.6 ± 13.26, which decreased to 11.42 ± 9.24 post-MSA, resulting in a mean reduction of 26.19 ± 14.37. In contrast, patients with a BMI > 35 (*n* = 11) had a mean pre-MSA GERD-HRQL score of 35.54 ± 18.88, which decreased to 17.27 ± 16.7 post-MSA, yielding a mean reduction of 18.27 ± 15.08. Objective postoperative assessment was reported in 3 studies with a trend toward improved DeMeester score compared to baseline (47.2 ± 22 vs. 24.3 ± 15).

## Discussion

The global rise of obesity has led to increased utilization of bariatric surgery, with LSG being the most common weight-loss procedure performed worldwide [27, 28]. The prevalence of GERD is up to 50% in patients with BMI > 30 [29]. Following LSG, pre-existing GERD can worsen or symptoms can develop de novo in 19% and 26.7% of the patients, respectively, possibly leading to esophagitis and Barrett’s esophagus [1, 30–34]. Post-sleeve GERD may also be associated with intrathoracic sleeve migration in up to 30% of the cases [2, 3]. Elevated intragastric pressure, altered geometry of the angle of His, disruption of the sling fibers, HH, and intrathoracic sleeve migration are implicated in the pathogenesis of post-LSG GERD [35–39].

First-line management with PPIs can alleviate symptoms in about 60–70% of the patients [32, 33], although dose escalation may be necessary and a number of medication-related side-effects have been reported in the long run [4, 31, 34]. For those patients who are refractory to medical therapy, current surgical options are limited and include HH repair and/or RYGB [40]. Some authors proposed a prophylactic sleeve-fundoplication as a primary procedure with promising results [41, 42]. However, this is not universally accepted and may be associated with a significant incidence of postoperative gastric necrosis and perforation [43, 44]. Given the lack of evidence-based clinical guidelines, it appears reasonable to err on the side of caution and perform a comprehensive anatomical and pathophysiological assessment to select the most appropriate surgical procedure for post-LSG GERD [45]. It has been shown that symptoms do not predict abnormal reflux burden or esophageal motility disorders in bariatric patients and that achalasia, esophagogastric junction outflow obstruction, and GERD can occur without specific symptoms [46, 47]. The role of HH repair and LES augmentation, either concurrently or after LSG, is still controversial due to the paucity of high-quality data. A randomized controlled trial comparing LSG with or without HH repair showed similar outcomes in terms of postoperative GERD burden [48]. Interestingly, a comparative analysis of the MBSAQIP database including 48 patients showed that MSA performed prophylactically at the time of sleeve or bypass did not increase operative time and length of stay and was safe in the short-term follow-up. However, it is not clear from this study whether a concurrent hiatal repair was performed [49].

**Fig. 2:**
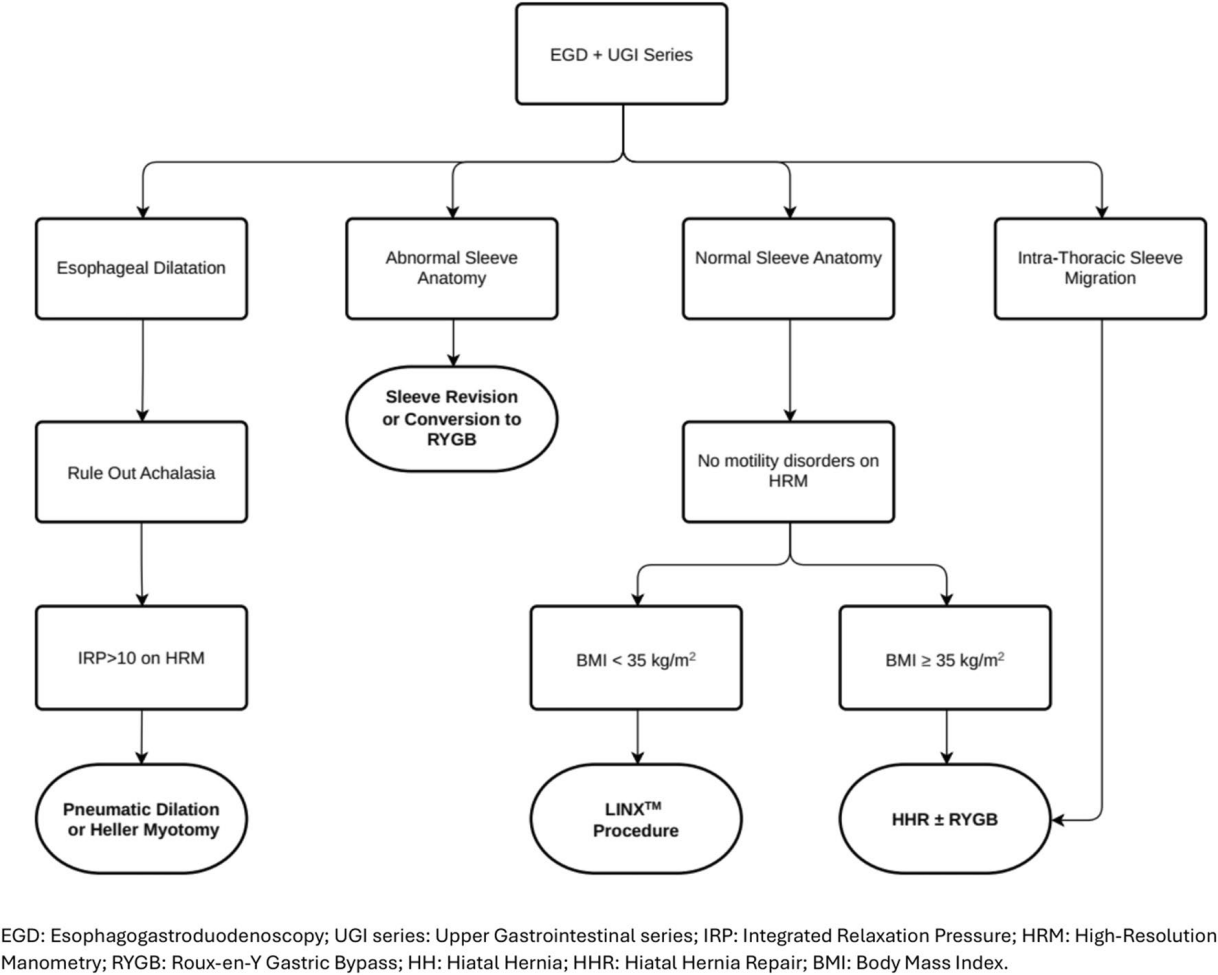
Proposed management algorithm for post-sleeve gastrectomy upper gastrointestinal symptoms

Based on the present review and the analysis of current literature, we propose a management algorithm for post-sleeve gastrectomy GERD (Fig. 2).

Patient selection for surgery is critical in this patient population, as GERD complications may sometimes result from sleeve morphology issues such as narrowed/twisted sleeve, stenosis at the incisura angularis, or fundus dilation [50–52]. Revision of the sleeve to a more tubular shape might be feasible, though it carries the risk of leaks due to increased intraluminal pressure and impaired blood supply [53]. Therefore, in patients with abnormal sleeve anatomy and/or significant weight regain after LSG, conversion to RYGB often remains the first-choice option [52]. However, this procedure is not well accepted by patients with optimal weight loss after LSG and may be associated with significant surgical and metabolic complication rates [54–57]. Last but not least, the gastric remnant becomes inaccessible for endoscopic screening [58].

Safety and effectiveness of the LINX™ procedure for GERD in non-bariatric patients has already been demonstrated in several studies. It is well known that implantation of the MSA device can augment the LES by inhibiting its effacement under challenges of intragastric pressure [12, 59–62]. Further, MSA has been shown to be as effective as fundoplication in controlling reflux symptoms, particularly regurgitation [63–66], with less gas-bloat symptoms and fewer issues with belching and vomiting [66]. Since 2007, the indications for use of the MSA device have gradually expanded to include large hiatal hernias and Barrett’s esophagus. It is now recommended that routine mediastinal dissection, esophageal mobilization, and cruroplasty should be incorporated in the LINX™ procedure [67–71]. Given the worldwide adoption of LSG and the increase in postoperative GERD rates, the LINX™ procedure could represent a viable alternative to RYGB [12, 18], but no randomized studies exist and high-quality reports on safety and efficacy are scanty [49]. Since the esophagogastric junction and the crura work synergistically in restoring the antireflux barrier, it seems logical to assume that both hiatal repair and LES augmentation with LINX™ procedure may represent a good option in patients with post-sleeve GERD and BMI < 35 kg/m^2^ or those patients who decline RYGB [40, 72]. However, clinical evidence remains limited and no conclusive guidelines are available yet.

This review examined 14 studies conducted between 2015 and 2024, involving a total of 109 patients, with a predominance of females (82.6%). The wide age range (25–73 years) and variability in BMI (21.1 to 51.9) underscore the diverse patient population undergoing this procedure. Among the studies included, only 37 patients had clearly identifiable baseline BMI and pre- and postoperative GERD-HRQL scores. A trend was noted toward greater improvement in GERD-HRQL scores for patients with a BMI < 35, although this did not achieve statistical significance, likely due to the small sample size. Moreover, the time interval between LSG and LINX™ placement varied significantly (7 to 124 months). This variability highlights the current heterogeneity in the management of post-LSG GERD in clinical practice and the lack of guidelines for objective patient assessment, follow-up, and timing of intervention [73].

It is noteworthy that outcomes of the index operation, such as the percent of excess weight loss, and objective investigations such as barium swallow studies, pH-impedance testing, and high-resolution manometry were infrequently reported in the studies analyzed for this review. Additionally, details regarding the extent of mediastinal dissection, the length of the intra-abdominal esophagus following crural repair, the type of crural repair performed, the sizing technique for selecting the appropriate MSA device, and the placement of the device through a window within the posterior vagus nerve were not provided. It is evident that lack of standardization of the surgical procedure may potentially increase complication rates [74, 75]. In this review, LINX-related adverse events were mostly transient and self-limiting, consistent with the majority of observational studies in non-bariatric patients. Further, the decrease in the GERD-HRQL scores and the fact that up to 76% of the patients were off or had a significantly reduced PPI use indicate improved quality of life. Despite the limited objective testing and absence of longitudinal follow-up data, overall patient satisfaction with the procedure was reported as good in 5 out of 14 studies. Unfortunately, no long-term safety and efficacy data for the LINX™ procedure are currently available for the bariatric surgical population [71].

### Study Limitations and Quality Assessment

This review has several limitations, including its retrospective design, potential selection bias, and small sample size. Moreover, the median follow-up was only 2 years, and outcome measures varied across studies. Since most patients had BMI values below 35, it remains unclear whether similar outcomes can be achieved in individuals with a BMI above 35. None of the studies reported obesity outcomes such as weight loss measured as percent of excess body weight or indicators of central obesity such as the waist-to-hip/height ratio. All included studies reported postoperative clinical (symptoms improvement) and PPI suspension rates but failed to report objective postoperative GERD assessment (i.e., endoscopy, HRM, and pH-impedance test). Furthermore, the predominant geographical concentration of studies in the USA may restrict the generalizability of the findings to other countries. Key technical details of the LINX™ procedure were also not disclosed in all papers (i.e., intraoperative sizing protocol). Another limitation is the lack of information on steroid use or specific postoperative regimens; future research should explicitly address this aspect, especially in patients with comorbidities like T2DM. Lastly, quality assessment indicated moderate to serious risks of bias in most studies, underscoring the need for cautious data interpretation and more rigorous research in this area. Therefore, the overall body of evidence remains frail due to the small number of patients, lack of objective postoperative assessments, and short follow-up time across most studies.

## Conclusion

This review suggests that magnetic sphincter augmentation may represent a feasible, safe, and less invasive option compared to RYGB for selected patients with post-sleeve GERD, especially those with a normalized or < 35 kg/m^2^ BMI and no evidence of associated esophageal motility disorders.

While recognizing that definitive conclusions cannot be drawn at present, we believe that well-designed, prospective, and long-term follow-up studies will clarify the role of the LINX procedure in this patient population.

## Supplementary Information

Below is the link to the electronic supplementary material.

Supplementary file1 (DOCX 109 KB)

Supplementary file2 (DOCX 23 KB)

## Data Availability

The data are available from the authors upon reasonable request.
