# Supplementary material for: Magnetic Sphincter Augmentation for Gastroesophageal Reflux After Sleeve Gastrectomy: A Systematic Review
Source: Obes Surg. 2024 Oct 15;34(11):4232–43. doi: 10.1007/s11695-024-07523-8 (PMC11541252; doi:10.1007/s11695-024-07523-8)
Supplement: Supplementary file 1 — Supplementary file1 (DOCX 109 KB) [file 11695_2024_7523_MOESM1_ESM.docx]

**Appendix 1: Search Strategy**

The research was conducted using the following databases, searching for all articles published up to May 1, 2024. The online databases/libraries, the search keys used, and the number of articles identified are listed below:

1. **EMBASE**
   - **Search Key**: (linx OR msa OR (magnetic AND sphincter) OR (magnetic AND sphincter AND augmentation)) AND (sleeve OR gastroplasty OR magenstrasse)
   - **Number of Articles Identified**: 98
2. **Web of Science (WOS)**
   - **Search Key**: (linx OR msa OR (magnetic AND sphincter) OR (magnetic AND sphincter AND augmentation)) AND (sleeve OR gastroplasty OR magenstrasse)
   - **Number of Articles Identified**: 53
3. **SCOPUS**
   - **Search Key**: (linx OR msa OR (magnetic AND sphincter) OR (magnetic AND sphincter AND augmentation)) AND (sleeve OR gastroplasty OR magenstrasse)
   - **Number of Articles Identified**: 65
4. **PubMed**
   - **Search Key**: (linx OR msa OR (magnetic AND sphincter) OR (magnetic AND sphincter AND augmentation)) AND (sleeve OR gastroplasty OR magenstrasse)
   - **Number of Articles Identified**: 32
5. **Cochrane Library**
   - **Search Key**: (linx OR msa OR (magnetic AND sphincter) OR (magnetic AND sphincter AND augmentation)) AND (sleeve OR gastroplasty OR magenstrasse)
   - **Number of Articles Identified**: 1
6. **Google Scholar**
   - **Search Key**: “linx” AND “sleeve gastrectomy”
   - **Number of Articles Identified**: 364
