# Supplementary material for: Magnetic Sphincter Augmentation for Gastroesophageal Reflux After Sleeve Gastrectomy: A Systematic Review
Source: Obes Surg. 2024 Oct 15;34(11):4232–43. doi: 10.1007/s11695-024-07523-8 (PMC11541252; doi:10.1007/s11695-024-07523-8)
Supplement: Supplementary file 2 — Supplementary file2 (DOCX 23 KB) [file 11695_2024_7523_MOESM2_ESM.docx]

| \| **Supplementary Table 1**. Quality assessment of the included Observational non-randomized studies (ROBINS-I tool). Each domain is evaluated with one of the following: Low, Moderate, Serious, and Critical. The categories of judgement for each study are low, moderate, serious, and critical risk of bias. \| \| --- \| | | | | | | | | |
| --- | --- | --- | --- | --- | --- | --- | --- | --- | --- |
| **Author, Year, Country, Study Design** | **Confounding Bias** | **Selection Bias** | **Classification Bias** | **Intervention Bias** | **Missing Data Bias** | **Measurement Bias** | **Reporting Bias** | **Bias** |
| Peine et al., 2024, Ret | Moderate | Moderate | Low | Low | Serious | Moderate | Low | Serious |
| Khaitan et al., 2023, PrO | Moderate | Moderate | Low | Low | Moderate | Moderate | Low | Moderate |
| Hawasli et al., 2023, Ret | Moderate | Moderate | Low | Low | Serious | Moderate | Low | Serious |
| Patel et al.,  2022, Ret | Moderate | Moderate | Low | Low | Moderate | Moderate | Low | Moderate |
| Bellorin et al., 2021, PrO | Moderate | Moderate | Low | Low | Moderate | Moderate | Low | Moderate |
| Broderick et al., 2020, Ret | Moderate | Moderate | Low | Low | Moderate | Moderate | Low | Moderate |
| Desart et al., 2015, Ret | Moderate | Moderate | Low | Low | Moderate | Moderate | Low | Moderate |
|  | | | | | | | | |

| **Supplementary Table 2**. Quality assessment of the included Case reports and Case series (Murad et al. protocol). Each domain is evaluated with one of the following: Low, Moderate, High. The categories of judgement for each study are low, moderate, high. | | | | | |
| --- | --- | --- | --- | --- | --- |
| **Author, Year, Country, Study Design** | **Selection** | **Ascertainment** | **Causality** | **Reporting** | **Bias** |
| Soler-Silva et al., 2023, CR | Moderate | Low | Moderate | Low | Moderate |
| Asti et al.,  2021, CR | Moderate | Low | Moderate | Low | Moderate |
| Ndubizu et al.,  2020, CR | Moderate | Low | Moderate | Low | Moderate |
| Ajabshir et al.,  2019, CR | Moderate | High | Moderate | Moderate | High |
| Hawasli et al.,  2017, CR | Moderate | Moderate | Moderate | Low | Moderate |
| Pixner et al.,  2022, CR | Moderate | Moderate | Moderate | Low | Moderate |
| Bona et al.,  2022, CR | Moderate | Low | Moderate | Low | Moderate |
